# Biomarkers of exposure in urine of active smokers, non-smokers, and vapers

**DOI:** 10.1007/s00216-023-04943-w

**Published:** 2023-09-25

**Authors:** D. Gallart-Mateu, P. Dualde, C. Coscollà, J. M. Soriano, S. Garrigues, M. de la Guardia

**Affiliations:** 1 Department of Analytical Chemistry, University of Valencia, Research Building, 50 Dr. Moliner Street, 16100-Burjassot, Valencia, Spain; 2 Foundation for the Promotion of Health and Biomedical Research in the Valencian Region, FISABIO-Public Health, Av. Catalunya, 21, 46020 Valencia, Spain; 3 GISP Grup d’Investigació en Salut Pública, Universitat Politècnica de Catalunya, Barcelona, Spain

**Keywords:** Smoker and vaper, Urine, Biomarker, E-cigarette, Organic compounds, Nicotine

## Abstract

The exposure to smoking related products has been evaluated through urine illness risk marker determination through the analysis of urine samples of smokers and vapers. Biomarkers and their metabolites such as *N*-acetyl-*S*-(2-cyanoethyl)-L-cysteine (CEMA), *N*-acetyl-*S*-(3,4-dihydroxybutyl)-L-cysteine (DHBMA), *N*-acetyl-*S*-[1-(hydroxymethyl)-2-propen-1-yl)-L-cysteine (MHBMA), *N*-acetyl-*S*-(3-hydroxypropyl)-L-cysteine (3HPMA), 2*R*-*N*-acetyl-*S*-(4-hydroxybutan-2-yl)-L-cysteine (HMPMA), and *N*-acetyl-*S*-(3-carboxy-2-propyl)-L-cysteine (CMEMA) together with nicotine and cotinine were identified and quantified by LC-HRMS and LC-MS/MS, and data found normalized to the creatinine level. One hundred two urine samples were collected from smokers, non-smokers, and vapers, spanning an age range from 16 to 79 years. Results obtained showed that CEMA was only detected in urine samples from smokers and MHBMA was in the same order of magnitude in all the urine samples analyzed. HMPMA was found in the urine of vapers at the same order of concentration as in non-smokers. 3HPMA in vapers was lower than in the urine of smokers, presenting an intermediate situation between smokers and non-smokers. On the other hand, DHBMA in vapers can reach similar values to those found for smokers, while CMEMA shows concentrations in the urine of vapers higher than in the case of non-smokers and traditional smokers, requiring new research to link this metabolite to the use of electronic cigarettes and possible alternative metabolomic routes. In general, this study seems to verify that traditional smoking practice constitutes a major source of carcinogenic chemicals compared with substitutive practices, although those practices are not free of potential harm.

**Graphical abstract:**

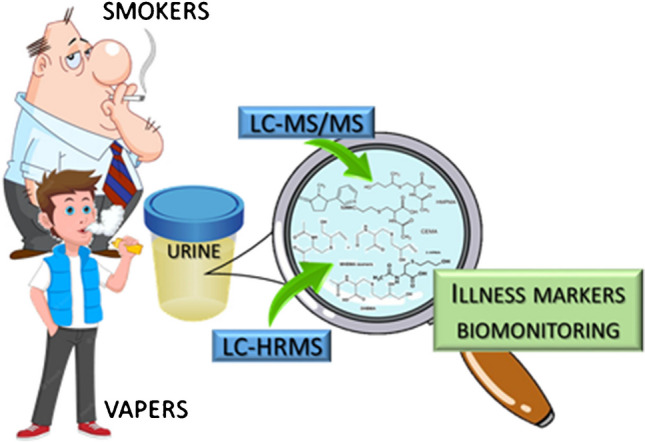

**Supplementary Information:**

The online version contains supplementary material available at 10.1007/s00216-023-04943-w.

## Introduction

Tobacco use, as a traditional practice in many countries around the world, is the main cause of death estimated for eight million people each year [1], and, in the case of Spain, it is estimated 69,000 deaths/year due to smoking [2]. Although the harmful effects of smoking are almost universally known, there were 933 million smokers worldwide in 2015. More than 80% of the world’s smokers live in low- and middle-income countries [3]. It is estimated that about two-thirds of lung cancer deaths worldwide are due to smoking [4]. Smoking is also a cause of cancers of the oral cavity, pharynx, larynx, esophagus, nasal cavity, pancreas, bladder, stomach, liver, kidney, ureter, cervix, colorectum, and ovary (mucinous), as well as myeloid leukemia [5, 6].

The smoking addiction results from the rapid delivery of nicotine to the brain, leading to its highly reinforcing effects [5]. However, the devastating effects of burning tobacco are associated to the combustion products. Responding to this situation, in the last decade, there has been an explosion of alternative smokeless tobacco products as alternative to traditional tobacco, such as electronic cigarettes or heat not burn tobacco products. However, research articles reporting the composition of the generated aerosols by this type of devices have evidenced the presence of toxic compounds, such as carbonyl compounds, tobacco-specific nitrosamines (TSNAs), acrolein, or acrylamide [7–11]. In the same way, toxics have been reported in vaping liquids of electronic cigarettes [9, 12, 13]. Although the presence of nicotine in the aerosol generated at low temperature in electronic cigarettes is considerable [7], the amount of toxins and carcinogens is lower when compared to traditional tobacco smoke [14]. Despite of this, some studies have reported cancer disease development due to the exposure to electronic cigarettes in animals [15] or DNA damage on electronic cigarette consuming [16].

On the other hand, long-term exposure to some environmental, occupational, and non-occupational toxicants, such as benzene, toluene, styrene, 1,3-butadiene, and acrylamide, has adverse health effects, including the development of cancer [17–22].

Tobacco smoke, and in the same way the e-cigarettes aerosols, is a relevant non-occupational source of exposure [6, 23]. The main component of tobacco smoke use to be nicotine nicotine, which is primarily metabolized to cotinine in the human liver via CYP2A6 [24, 25]. Cotinine, the main metabolite of nicotine [26], is often used as a biomarker of tobacco exposure in humans [27–30] due to its higher concentration in blood (250–300 vs 10–50 ng/mL) and longer residence time than nicotine (16 vs 2 h) [27, 31]. However, the organism metabolizes the toxic compounds present in the smoke/vaping aerosols. Following inhalation, toxic compounds present in the smoke may undergo initial biotransformation (phase I metabolism) in the liver, to active, oxygenated, electrophilic intermediates, primarily due to oxidative action of cytochrome P450 enzymes. These intermediates are believed to be reactive species able of reacting with DNA and responsible for the genotoxicity associated with the parent compounds and subjected to the “mercapturic acid pathway” (phase II metabolism). During this biotransformation, catalyzed by the enzyme glutathione transferase, the electrophilic compound is deactivated by conjugation with glutathione, an endogenous tripeptide made up of glutamic acid, cysteine, ​​and glycine. By means of other enzymatic reactions, the glutamic acid and, subsequently, the glycine are removed. The remaining cysteine ​​conjugate is N-acetylated and eventually excreted in the urine as mercapturic acid [32, 33]. Thus, urinary mercapturic acids may be useful for assessing exposure to toxicants from both traditional tobacco and smokeless tobacco devices as well as many other exposure sources [32, 34].

Taking into account the aforementioned factors, the aim of this work concerns the comparison of some specific acrolein, acrylonitrile, benzene, and crotonaldehyde metabolite concentrations in urine, cited in literature as health risk markers [35] together with nicotine and cotinine in both, users of traditional burn tobacco and former smokers using vaping systems. Non-smokers urine was employed also as reference.

## Material and methods

### Reagents, standards, and samples

N-acetyl-S-(2-cyanoethyl)-L-cysteine ammonium salt >98% (CEMA, CAS: 74514-75-3), N-acetyl-S-(3,4 dihydroxybutyl)-L-cysteine mixture of diastereomers >95% (DHBMA, CAS: 144889-50-9), and (R,S)-N-acetyl-S-[1-(hydroxymethyl)-2-propen-1-yl)-L-cysteine + (R,S)-N-acetyl-S-(2-hydroxy-3-buten-1-yl)-L-cysteine 1:1 mixture >95% (MHBMA, CAS: mixture of 144889-51-0 and 159092-64-5) were obtained from Toronto Research Chemicals (Toronto, ON, Canada). N-acetyl-S-(3-hydroxypropyl)-L-cysteine sodium salt >98% (3HPMA, CAS: 14369-42-7), 2R-N-acetyl-S-(4-hydroxybutan-2-yl)-L-cysteine mixture of diastereomers >95% (HMPMA, CAS: 33164-64-6), and N-acetyl-S-(3-carboxy-2-propyl)-L-cysteine disodium salt mixture of diastereomers >98% (CMEMA, CAS: 1041285-62-4) were obtained from TLC Pharmaceutical Standards (New Market, ON, Canada). (-)-Nicotine >99% from Sigma-Aldrich (St. Louis, MO, USA) and 1 mg mL^−1^ cotinine standard solution in methanol from Cerilliant Corporation (Round Rock, TX, USA) were employed. On the other side, 100 µg mL^−1^ nicotine-d_4_ standard solution in acetonitrile from Sigma-Aldrich (St. Louis, MO, USA) was used as nicotine and cotinine internal standard. Terfenadine and Val-Tyr-Val from Sigma Life Sciences (St. Louis, MO, USA), triallyl phosphite from Alfa Aesar Thermo Fischer (Kandel, Germany) and sulfaguanidine, sulfadimethoxine, reserpine, caffeine, and acetaminophen obtained from Sigma-Aldrich (St. Louis, MO, USA) were employed as internal standards for LC-MS/MS in metabolite determination.

Acrylonitrile (≥ 98%) provided by Sigma-Aldrich (St. Louis, MO, USA) and crotonaldehyde (>99%) mixture of isomers from Merck (Darmstadt, Germany) were employed in the volatile compounds experimental part. Toluene-d_8_ (>99.9%) from Sigma-Aldrich (St. Louis, MO, USA) and acetonitrile for GC trace residue analysis (≥ 99.9%) from Scharlau (Barcelona, Spain) were employed as internal standards.

Methanol (LC-MS grade), acetonitrile (LC-MS quality), and the buffer constituents, acetic acid and ammonium formiate, were provided by VWR Chemicals (Radnor, PA, USA) and Scharlau (Barcelona, Spain). Water employed with a maximum resistivity of 18.2 MΩ was obtained from a Milli-Q Millipore system (Bedford, MA, USA).

Sterile flasks were used to collect the first morning urine samples of 102 non-affected by renal affections subjects, including 14 smokers, 25 non-smokers, and 63 declared vapers to determine the presence and quantify the target metabolites. Candidates, men and women aged between 16 and 79 years were selected taking into account their former smoking practice and present vaping habits. In general, volunteers smoked for 4–30 years before move to vaping and used vaping systems during the last 4 to 146 months. In all cases, the donors were informed of the objective of the study, and each volunteer was provided with an informed consent document including a confidentiality agreement between the university and the participants.

Once the samples were received, aliquots of 10 mL were taken and together with the original samples were frozen until analysis.

### Sample population

Sampling process was performed according the guidelines of the ethics committee of the University of Valencia, verification code X0H21EQATBAG6TVF (see Figure S1). Table S1 indicates the characteristics of 102 subjects included in this study. As can be seen, a set of 51 male and 51 female was considered, spanning the age range from 20 to 78 years for men and from 16 to 75 years for women. Urine samples were obtained from 63 vapers (35 male and 28 female), 14 smokers (6 male and 7 female), and 25 non-smokers (9 male and 16 female) as control group. Creatinine values, obtained by the Jaffé reaction, were employed to normalize the urinary biomarker levels. It was found creatinine data which varied from 0.31 to 2.83 g L^−1^_urine_, being in concordance with levels found in literature for healthy individuals [34]. However, the creatinine reference approach is not advocated for urine samples with very low (< 0.3 g/l) or high (> 3 g/l) concentrations. Only for three samples, the creatinine contents were higher than 3 gL^−1^ as it has been noticed in Table S1.

### Creatinine analysis

Creatinine was used to normalize the data of target analytes in order to minimize the effect of characteristics and habits of the volunteers. Creatinine determination in urine samples was performed using a Linear Kroma autoanalyzer (Holliston, MA, USA). The analysis methodology is based on the colorimetric determination of creatinine using a urine volume of 20 µL by formation of a chromogen (the Janovsky complex) by reaction with alkaline picrate through the Jaffé reaction [36]. The employed methodology allowed the determination of creatinine in urine samples down to concentrations of 0.01 g L^−1^.

### LC-MS/MS procedure

#### Nicotine and cotinine determination in samples

Sample analysis was performed using a Thermo Scientific (Waltham, MA, USA) Vanquish UHPLC chromatograph with a Thermo Scientific (Waltham, MA, USA) TSQ Altis triple quadrupole mass detector. For the separation of the analytes, a Hypersil GOLD C18 column, 1.9 μm (150 × 2.1 mm), provided by Thermo Scientific (Waltham, MA, USA), was used, together with a gradient method with two mobile phases, one of 0.1% acetic acid in ultrapure water (A) and another of 0.1% acetic acid in LC-MS grade MeOH (B). A 0.3 mL min^−1^ constant flow was applied. In order to perform the chromatography separation, the following gradient program was used: at the initial time, the mobile phase composition was 90% (A) decreasing till 75 % (A) at 6 min, increasing till 90 % (A) at 6.5 min, and maintaining these conditions till the end of the chromatogram (10 min).

The detection of the analytes was carried out using positive ionization (ESI^+^) and applying a voltage of 3500 V. The column temperature was set at 35°C, the injections were measured in cycles of 0.8 s, and the injection volume was 10 µL. The m/z transitions used for the identification and quantification of the analytes were those indicated in Table S2.

For quantification of nicotine and cotinine, a concentrated multicomponent solution in methanol was prepared with the commercial standards. Calibration solutions were prepared in the concentration range of 190–3800 ng mL^−1^ in the case of nicotine and 150–3000 ng mL^−1^ in the case of cotinine. The calibration curve was prepared by adding 100 μL of urine (urine blank), 10 μL (2000 ng mL^−1^) of internal standard, and an appropriate volume of multicomponent standard solution, being completed with ultrapure water up to a 1 mL final volume.

1 mL of samples, properly diluted, were spiked with 10 μL of internal standard and analyzed.

In order to study the precision and accuracy of the nicotine and cotinine determination in urine samples, urine blanks from a non-smoker were spiked per triplicate with a multi-analyte standard at various analyte concentration levels spanning a concentration range between 150 till 3800 ng mL^−1^.

#### DHBMA, MHBMA, CEMA, 3-HPMA, CMEMA, and HMPMA analysis procedure

A Vanquish UHPLC chromatograph from Thermo Scientific (Waltham, MA, USA) was used with a triple quadrupole mass detector TSQ Altis from Thermo Scientific (Waltham, MA, USA). For the separation of the analytes, a Hypersil GOLD C18 column, 1.9 μm (150 × 2.1 mm), provided by Thermo Scientific (Waltham, MA, USA), was employed using a gradient of two mobile phases, ammonium formate (5mM) in 0.1% acetic acid in ultrapure water (A) and acetonitrile in HPLC-MS quality (B). The column temperature was 40°C. To perform the separation, the applied mobile phase gradient was as follows: 99.5 % (A) from the beginning to 0.5 min, then decreasing to 70 % (A) till 2 min, maintaining 70% (A) till 7 min, followed by decreasing to 0 % (A) at 8 min and maintaining 0% till 12 min, and finally, increasing the proportion of mobile phase A to 99.5 % at 12.1 min and maintaining this proportion till the end of the program (18 min).

The detection of analytes was carried out using positive ionization (ESI^+^) by applying a voltage of 3500 V and negative ionization (ESI^-^) by applying a voltage of 3300 V. The injections were measured in cycles of 0.8 s, and the injection volume was 10 µL. The m/z transitions used for the identification and quantification of the analytes and the ionization mode are indicated in Table S2.

A concentrated multi-analyte solution in water was prepared from commercial standards of the analytes. The calibration solutions were prepared in the concentration range between 9 and 750 ng mL^−1^ and made by mixing 100 μL of non-smokers urine (urine blank), 20 μL of 8 µg mL^−1^ terfenadine, Val-Tyr-Val, triallyl phosphite, sulfaguanidine, sulfadimethoxine, reserpine, caffeine and acetaminophen multi-internal standard solution, and the appropriate volume of multi-analyte standard solution to reach the required concentration. Ultrapure water was added to reach a final volume of 1 mL.

One milliliter of properly diluted urine samples were spiked with 20 μL of 20 μL of terfenadine, Val-Tyr-Val, triallyl phosphite, sulfaguanidine, sulfadimethoxine, reserpine, caffeine, and acetaminophen multi-internal standard solution of an adequate concentration and analyzed.

The precision and accuracy of analyte determination were established from three independent replicates of non-smoker urine blanks spiked at concentrations between 10 and 750 ng mL^−1^ and analyzed as unknown samples.

### HS-GC/MS urine analysis

HS-GC-MS was used to determine the presence of acrylonitrile and crotonaldehyde due to their high volatility.

#### Acrylonitrile analysis

An Agilent Technologies 5975A GC System chromatograph (Palo Alto, CA, USA) equipped with a Zebron ZB-5MS 5% capillary column (30 m, 0.32 mm, 0.25 μm) and an Agilent Technologies 5975C inert XL EI/CI MSD triple axis simple quadrupole mass detector was used.

Acrylonitrile stock solutions were prepared in methyltetrahydrofuran (MeTHF) from pure acrylonitrile stock (>98%) (Darmstadt, Germany). As an internal standard, a solution of 1000 µg mL^−1^ of acetonitrile in MeTHF was used, prepared from a 99.9% HPLC-grade acetonitrile provided by Scharlab (Barcelona, ​​Spain). MeTHF 99% supplied by Pennakem (Memphis, TN, USA) was used to prepare all solutions. HS analysis was performed using glass vials with an internal volume of 10 mL encapsulated with PTFE-silicone seals. Samples were injected into the chromatographic system using an Agilent Technologies 7697A Headspace sampler autosampler.

For the preparation of the calibration curve, solutions of the analyte with concentrations from 100 to 800 ng mL^−1^ were used, allowing a working range between 0.5 and 4 ng of acrylonitrile in urine samples. Ten microliters of the solutions of corresponding concentration were introduced into glass vials of 10 mL volume together with 5 µL of the 1000 µg mL^−1^ acetonitrile internal standard solution. Vials were capped and tested directly. Direct vaporization of acrylonitrile and internal standard was carried out at 90°C for 10 min. The gaseous fraction of the headspace was introduced through an injection loop of 1 mL volume at 95°C in split mode with a 1:8 ratio in the injector at 180°C, using a flow constant 1.3 mL min^−1^ of helium as carrier gas. Chromatographic separation was performed using an initial temperature of 60°C maintained for 6 min and increased with a ramp of 15°C min^−1^ until reaching 110°C. The transfer line and ionization source temperatures were 280 and 276°C, respectively.

The ionization of the analytes in the detector was performed using the electron impact (EI) mode at 70 eV, and the selected ion monitoring (SIM) was used for the acquisition of the signals of the analytes, registering the masses 53.1 m/z and 41.1 m/z for the quantification of acrylonitrile and acetonitrile, respectively.

Twenty microliters of urine samples was weighed with accuracy per triplicate with an analytical balance, and 5 μL of the 1000 μg mL^−1^ internal standard solution was added, encapsulated, and analyzed together with the standards.

#### Crotonaldehyde analysis

The determination of crotonaldehyde in urine samples was carried out according to the procedure proposed by Guo et al. [37]. For this purpose, an Agilent Technologies 5975A GC System chromatograph (Palo Alto, CA, USA) equipped with a Zebron ZB 5MS 5% capillary column (30 m, 0.32 mm, 0.25 μm) and an Agilent Technologies 5975C inert XL EI/CI MSD triple axis simple quadrupole mass detector was used.

Crotonaldehyde standard solutions were prepared in acetone from pure crotonaldehyde (≥99%) (Darmstadt, Germany). As an internal standard, a solution of deuterated toluene (toluene-d_8_) in acetone was used, prepared from deuterated toluene 99.9% Merck (Darmstadt, Germany). Acetone ≥99.9% for GC residue analysis provided by Scharlau (Barcelona, ​​Spain) was employed to prepare all solutions. HS analysis was performed using glass vials with an internal volume of 10 mL encapsulated with PTFE-silicone seals. Samples were injected into the chromatographic system using an Agilent Technologies 7697A Headspace sampler autosampler. For the preparation of the calibration curve, solutions of the analyte with concentrations of 300 to 2500 ng mL^−1^ were used, allowing the determination of crotonaldehyde in a range between 6 and 50 ng in samples. Five microliters of the standard solution with an adequate concentration was placed in glass vials of 10 mL volume together with 5 µL of the internal standard solution. The vials were capped and analyzed directly. Direct vaporization of acrylonitrile and internal standard were carried out at 90°C for 10 min. The gaseous fraction of the headspace was introduced through an injection loop of 1 mL volume at 95°C in split mode with a 1:8 ratio in the injector at 180°C, using a flow constant 1.3 mL min^−1^ of helium as carrier gas. Chromatographic separation was performed using an initial temperature of 40°C maintained for 2 min and increased with a ramp of 2°C min^−1^ until reaching 80°C. The transfer line and ionization source temperatures were 280 and 276°C, respectively.

The ionization of the analytes in the detector was performed using the electron impact (EI) mode at 70 eV, and the selected ion monitoring (SIM) was recorded at masses 41 m/z and 98 m/z for the quantification of crotonaldehyde and deuterated toluene, respectively.

The urine samples were weighed in triplicate on an analytical balance, placing 20 μL of each sample in glass vials with a volume of 10 mL. Five microliters of the internal standard solution were added, encapsulated, and analyzed in the same way as standards.

### Statistical treatment

Descriptive statistics were calculated by employing the Sigma-Plot software package (Systat Software Inc., Chicago, IL, USA). Parameters as average and standard deviation, median, 95% confidence interval, minimum and maximum values, and the corresponding percentiles were calculated for each sample population and target analytes.

## Results and discussion

### Analytical features of employed methodologies

The supplementary material provides data about the method validation of employed procedures.

#### LC-MS/MS analysis of nicotine and cotinine

The linearity of the method was established from the calibration curves specified in the “Nicotine and cotinine determination in samples” section prepared in non-smokers urine. In all cases, satisfactory determination coefficients (*R*^2^) were found, finding values between 0.991 and 0.992. The limits of detection (LOD) and of quantification (LOQ) were determined as 3 and 10 times the intercept deviation divided by the slope. LOD values from 45 to 47 ng mL^−1^ and LOQs between 134 and 144 ng mL^−1^ were found for target analytes. Table S3 indicates the linearity and the LODs and LOQs values found.

Precision and accuracy of the analytical methodology were established from the recovery and RSD (%) of data corresponding to spiked urine (see Table S3). As can be seen, recovery values ranged between 93 and 104%, while RSD (%) values were in the range of 3 and 9%.

#### LC-MS/MS analysis of DHBMA, MHBMA, CEMA, 3-HPMA, CMEMA, and HMPMA

The method was validated in the same terms indicated before. The linearity provided determination coefficients (*R*^2^) between 0.990 and 0.998. The limits of detection (LOD) and quantification (LOQ) were from 2.8 to 3.1 ng mL^−1^ and LOQs between 9 and 11 ng mL^−1^. Table S4 indicates the linearity and the values obtained for the LODs and LOQs of the analytes studied. Precision and accuracy of the analytical methodology were established from the recovery study and RSD (%) of spiked urine. Table S4 indicates values obtained. As can be seen, recoveries ranged between 87 and 104% and RSD (%) values lower than 7% were found.

#### HS-GC/MS analysis of acrylonitrile and crotonaldehyde

The acrylonitrile determination method was validated in terms of linearity, limits of detection and quantification, precision, and accuracy. The linearity was established from the calibration curves of the analytes in the concentration range between the limit of quantification and the loss of linearity in the analytical signal. In all cases, average determination coefficients (*R*^2^) of 0.9951 were obtained. The limit of detection (LOD) and of quantification (LOQ) were determined from the calibration expressions as 3 and 10 times the intercept divided by the slope, obtaining values of 0.33 ng and 1.01 ng for LOD and LOQ, respectively. Table S3 indicates the linearity and the values obtained for the LOD and LOQ. Precision and accuracy were established from the RSD (%) values and recovery, respectively. Urine blanks from a non-smoker were spiked with a standard solution prepared in water at different acrylonitrile concentration levels and analyzed as unknown samples. Table S3 indicates the recovery and RSD (%) values obtained. The relative standard deviation values were lower than 11%, and the accuracy values found in a range between 84 and 99%.

Crotonaldehyde determination method has been validated in terms of linearity, limits of detection and quantification, precision, and accuracy. The linearity was established from the calibration curves of the analytes in a concentration range between the limit of quantification and the loss of linearity in the analytical signal. In all cases, average determination coefficients (*R*^2^) of 0.9951 were obtained. The limit of detection (LOD) and limit of quantification (LOQ) were determined from the calibration expressions as 3 and 10 times the intercept divided by the slope. A LOD value was established in 1.4 ng and the LOQ was 4.6 ng. Table S3 shows the linearity and the values obtained for the LOD and LOQ. The accuracy and precision of the analytical methodology were established from the recovery and RSD (%) values obtained. Urine blanks from non-smokers were spiked with a standard solution prepared in water at different crotonaldehyde concentration levels and analyzed as unknown samples. As can be seen, the RSD values were lower than 4%, with the recovery values which were in a range between 97 and 108%.

### Analysis of urine samples

#### Nicotine and cotinine content in urine

**Fig. 1:**
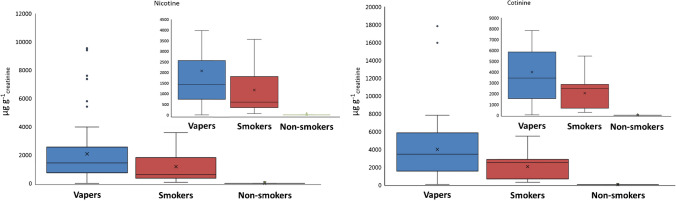
Box plot of nicotine and cotinine concentrations found in vapers, smokers, and non-smokers urine. Note: insets show data excluding anomalous

Table 1 indicates the statistical analysis related to the concentrations found in urine. Nicotine concentration ranged from 80 to 9543 µg g^−1^_creatinine_ for vapers consuming nicotine refilling solutions, while cotinine concentrations ranged from 302 to 17820 µg g^−1^_creatinine_ (see Table S5 for statistics related to the concentrations found in urine). In all cases, cotinine levels reach higher values than the nicotine concentrations, thus indicating the nicotine metabolism to cotinine (Fig. 1). On the other hand, nicotine-free vapers eliminate nicotine from their organism being detected very low concentrations of cotinine in their urine. Nicotine and cotinine concentrations found in urine of active smokers were from 60 to 3562 µg g^−1^_creatinine_ and from 276 to 5463 µg g^−1^_creatinine_, respectively. It can be seen that the nicotine/cotinine ratio variates for smokers and vapers.

**Table 1:** Comparison of the descriptive statistics of nicotine and cotinine concentrations expressed as µg g_creatinine_, in urine from non-smokers, smokers, and vapers

		Average ± SD	Median	95% conf. interval	Min value	5th percentile	25th percentile	75th percentile	95th percentile	Max. value
Nicotine	Non-smokers	-	-	-	-	-	-	-	-	-
	Smokers	1170 ± 1170	604	611	60	175	370	1648	3451	3565
	Vapers	2100 ± 2100	1479.5	519	80	159.5	854.5	2529.5	7375	9543
Cotinine	Non-smokers	-	-	-	-	-	-	-	-	-
	Smokers	2050 ± 1450	2482.5	749	276	324	794.5	2811.5	3783.5	5463
	Vapers	4000 ± 3300	3430	809	36	340	1770	5780	7791	17820

Additional metabolomic studies should be required, as it has indicated recently by Hsiao et al. [38] in order to study possible differences in the metabolic route of nicotine in both populations. However, this study does not take into account the differences in the metabolism between individuals, and relative values to creatinine must be considered to avoid individual variabilities.

Furthermore, neither nicotine nor cotinine were detected in non-smokers not living with smokers. However, it must be highlighted the case of passive smokers. Nicotine and cotinine concentrations were detected in the initial steps of this study in urine of non-smoker living with active smokers at concentrations of 84 and 90 µg g^−1^_creatinine_ and 64 to 74 µg g^−1^_creatinine_, respectively (see Table S5). These concentrations decreased until non-detected levels with the exposition cessation.

#### Risk exposure marker determination: analysis of acrylonitrile and crotonaldehyde

Acrylonitrile and crotonaldehyde, as selected risk exposition markers, were determined in urine samples. Data obtained were lower than the respective methodology limit of detection (see Table S3), being non-detected and thus indicating the completely metabolization of these analytes.

#### Metabolite content in urine samples

Table 2 indicates the descriptive statistical analysis related to the concentration found of the targeted molecules in the analyzed urine samples.

**Table 2:** Comparison of the descriptive statistics of studied metabolites concentrations expressed as µg g_creatinine_, in urine from non-smokers, smokers, and vapers

		Average ± SD	Median	95% conf. interval	Min value	5th percentile	25th percentile	75th percentile	95th percentile	Max. value
3HPMA	Non-smokers	610 ± 570	365.5	223	167	210	267	724	1376	2729
	Smokers	2210 ± 1275	1852	667	735	810.5	1286	2814	4480	4995
	Vapers	900 ± 700	761.5	168	172	224.5	463	1197	2587	3037
CEMA	Non-smokers	-	-	-	-	-	-	-	-	-
	Smokers	180 ± 80	169	52	41	53.5	102.5	262.5	302.5	370
	Vapers	-	-	-	-	-	-	-	-	-
HMPMA	Non-smokers	420 ± 460	252	185	76	127	195	405.5	1663	1829
	Smokers	2970 ± 1600	2960.5	833	881	945	1448	4159	5442	5650
	Vapers	600 ± 800	368.5	199	<LOQ	37.5	233	494	1461	5388
MHBMA	Non-smokers	95 ± 45	88.5	19	35	36.8	66.25	105.5	183.4	194
	Smokers	100 ± 40	107	23	37	43	64	137.5	154	166
	Vapers	130 ± 80	110	19	31	44.5	79.5	157	303	396
CMEMA	Non-smokers	800 ± 715	572.5	286	114	145	373	913.5	2372	3020
	Smokers	1630 ± 1985	863	1039	116	163	373	1809.5	6066	6102
	Vapers	2250 ± 2150	1459.5	526	213	337.5	820	370	5653	10,989
DHBMA	Non-smokers	350 ± 225	306	90	144	151	181.5	394.5	893	977
	Smokers	475 ± 260	444	137	53	123	270	724	826.5	855
	Vapers	400 ± 200	373	49	58	112	286	492	900	961

3HPMA, a metabolite of acrolein, was found in the three sample classes evaluated, varying the concentrations range from non-detected to 2868 µg g^−1^_creatinine_ for vapers’ urine, from 735 till 3106 µg g^−1^_creatinine_ for smokers, and from 167 to 2729 µg g^−1^_creatinine_ for non-smokers.

HMPMA and CMEMA, both crotonaldehyde metabolites, were found in vapers’ urine from non-detected to 2588 µg g^−1^_creatinine_, while a range from 881 to 4527 µg g^−1^_creatinine_ in smokers and from 76 to 1829 µg g^−1^_creatinine_ in non- smokers were determined. In the same way, CMEMA was found between 213 till 10989 µg g^−1^_creatinine_ in vapers, from 116 to 6102 µg g^−1^_creatinine_ in smokers, and from 76 till 3020 µg g^−1^_creatinine_ in non-smokers.

On the other hand, acrylonitrile/1.3-butadiene metabolites DHBMA and MHBMA were detected in all analyzed samples. DHBMA concentrations in vapers’ urine samples were from 58 till 961 µg g^−1^_creatinine_, while concentrations from 53 to 855 µg g^−1^_creatinine_ and 144 to 977 µg g^−1^_creatinine_ were found for smokers and non-smokers, respectively. In the same way, MHBMA concentrations spanned values from 36 µg g^−1^_creatinine_ for all the urine classes till 396, 166, and 194 µg g^−1^_creatinine_ for vapers, smokers, and non-smokers, respectively. CEMA acrylonitrile and acrolein metabolite were only detected in smokers samples, in a concentration range from 41 to 266 µg g^−1^_creatinine_.

#### Comparison of metabolite concentrations in smokers, non-smokers and vapers’ urine

##### Acrolein metabolites, 3HPMA

**Fig. 2:**
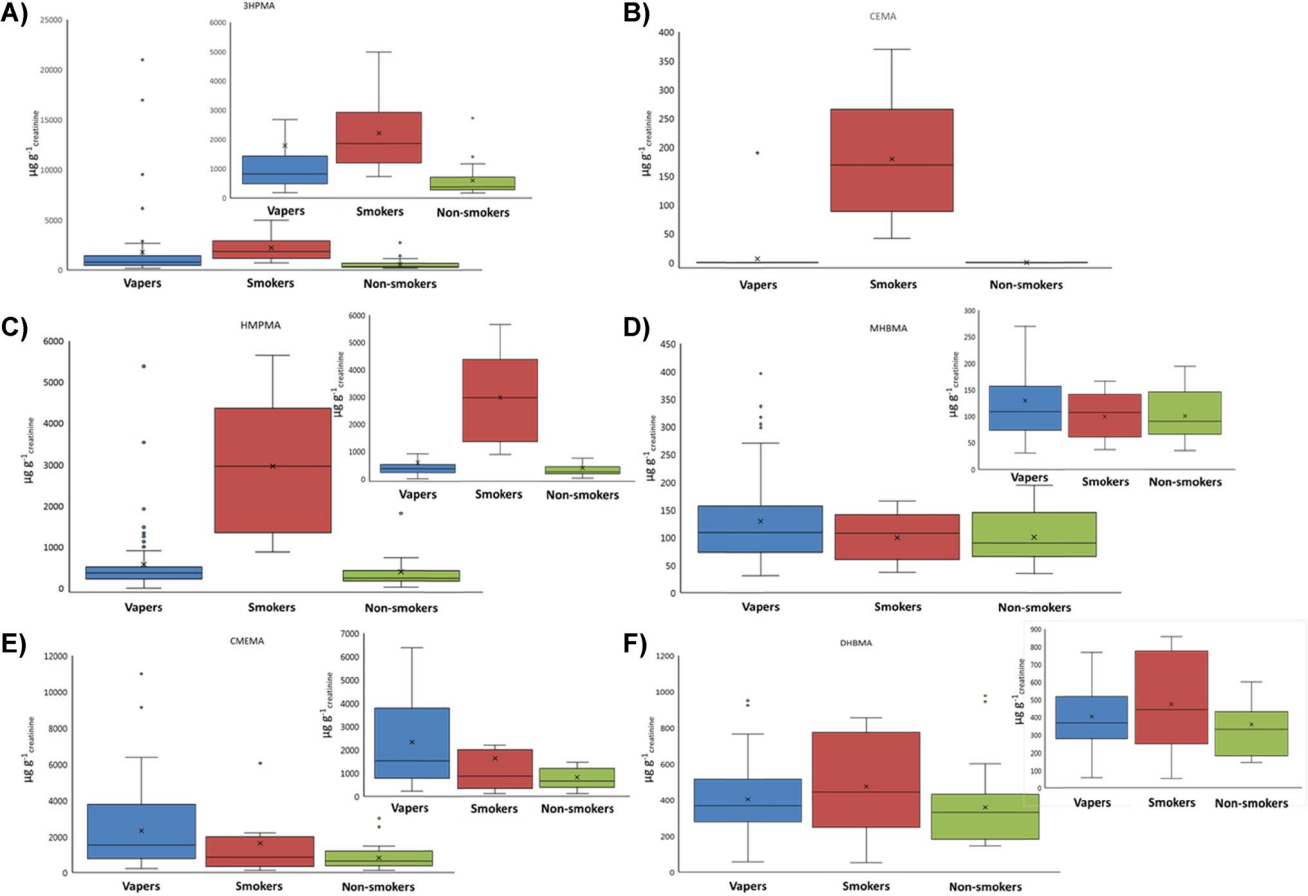
Box plot of 3HPMA (**A**), CEMA (**B**), HMPMA (**C**), MHBMA (**D**), CMEMA (**E**), and DHBMA (**F**) concentrations found in vapers, smokers, and non-smokers urine. Note: insets show data excluding anomalous

Figure 2A indicates the comparison of 3HPMA concentrations, expressed in µg g^−1^_creatinine_, in the urine samples of vapers, smokers, and non-smokers.

As can be seen, four urine samples (6, 32, 57, and 83) showed abnormal high levels of 3HPMA. Regarding the rest of samples, it was observed that vaper levels were usually lower than in the case of smokers. Non-smokers had lowest concentrations, being the values found in agreement with the literature [39–42]. Furthermore, 3HPMA values for smokers and vapers were consistent with those reported by Higashi et al. [43] and Bjurlin et al. [44], respectively.

##### Crotonaldehyde metabolites, HMPMA, and CMEMA

Figure 2C indicates the comparison of HMPMA concentrations found in urine of vapers, smokers, and non-smokers. As general trend, smokers’ urine samples show the highest concentrations of HMPMA, with the exception of sample 6 and 83, both belonging to candidates suspected to alternate vaping with traditional tobacco use. This behavior is in good agreement with the reported data for dual users [45]. On the other hand, HMPMA values for non-smokers agree with those found in the literature [46] and are the same order of magnitude in urine of non-smokers and vapers [47].

Figure 2E shows the comparison of CMEMA concentrations found in urine of vapers, smokers, and non-smokers. It can be seen that concentrations in vapers’ urine ranged between 213 and 10,990 µg g^−1^_creatinine_. These concentrations are higher than those reported by Frigerio et al. [48] for vapers’ urine, which ranged from 154 to 542 µg g^−1^_creatinine_. On the contrary, non-smokers and smokers urine show in general CMEMA concentrations in the same order of magnitude than that reported in the literature [46, 49]. So, although CMEMA is a secondary metabolite of crotonaldehyde, the behavior found in vapers’ urine must be studied from a metabolomic viewpoint, especially when data obtained for HMPMA in vapers are at the same level than in non-smokers.

##### Acrylonitrile metabolites, MHBMA, DHBMA, and CEMA

Figure 2D indicates the comparison of MHBMA concentrations found in urine from vapers, smokers, and non-smokers. The concentrations of MHBMA in vapers’ urine were of the same order of magnitude or slightly higher than those found in smokers and non-smokers ones. It is reported that MHBMA can be from exogenous sources, being reflected by literature its presence in non-smokers. Thus, MHBMA concentrations found in urine of non-smokers and vapers were in good agreement with those found in literature [50].

Similar concentrations of DHBMA were found for the three groups of urine samples (see Fig. 2F). The concentration found in vapers compared with those of smokers and non-smokers were similar, being this behavior previously reported [51].

CEMA was only detected on smokers and non-smokers exposed to burned tobacco (Fig. 2B). In this sense, those results were consistent with literature, being the CEMA concentrations in non-smokers and vapers’ urine close to the quantification limits or lower than this limit, and reported concentration in smokers urine samples are about two order of magnitude higher than for non-smokers or vapers [46].

## Conclusions

The present study has evidenced preliminary results on the prevalence of some metabolites of exposure toxicant markers related to diseases and cancer development in the urine of vapers, who in the past were strong smokers. It can be seen that CEMA metabolite is characteristic of tobacco smoke exposure/consumption, while the levels of HMPMA and MHBMA in urine from vapers are in the same order of magnitude than those found for smokers or non-smokers. On the other hand, DHBMA in urine from vapers can reach similar values to those found for smokers. However, CMEMA shown concentrations in the urine of vapers are higher than those found for non-smokers and smokers. This fact can require a new research to link this metabolite with the use of electronic cigarettes and possible alternative metabolomic routes. In the spite of the potential presence in toxic substances in electronic cigarette aerosols, there is not enough data regarding the short- and long-term health effects of e-cigarettes, including cancer risk. In this sense, the electronic cigarettes cannot be considered a safe alternative to the traditional tobacco consumption.

### Supplementary Information

Below is the link to the electronic supplementary material.

Supplementary file1 (DOCX 540 KB)
