# Supplementary material for: Biomarkers of exposure in urine of active smokers, non-smokers, and vapers
Source: Anal Bioanal Chem. 2023 Sep 25;415(27):6677–88. doi: 10.1007/s00216-023-04943-w (PMC10598069; doi:10.1007/s00216-023-04943-w)

**BIOMARKERS OF EXPOSURE IN URINE OF ACTIVE SMOKERS,**

**NON-SMOKERS AND VAPERS**

D. Gallart-Mateu*^a^*, P. Dualde*^b^*, C. Coscollà*^b^*, J. M. Soriano*^c^*, S. Garrigues*^a^*, M. de la Guardia*^a^**

*^a^* Department of Analytical Chemistry , University of Valencia, Research Building, 50 Dr. Moliner Street, 16100-Burjassot, Valencia, Spain

*^b^* Foundation for the Promotion of Health and Biomedical Research in the Valencian Region, FISABIO-Public Health, Av. Catalunya, 21, Valencia, 46020, Spain.

*^c^* GISP Grup d’Investigació en Salut Pública, Universitat Politècnica de Catalunya.

**Supplementary material.**

| ***Table S1.*** Sample population included in the study together with their gender, type of nicotine consumption system used, smoking time, use of an alternative practice to traditional tobacco and creatinine concentration in urine. | | | | | | | | | | | | | | | | | |  |
| --- | --- | --- | --- | --- | --- | --- | --- | --- | --- | --- | --- | --- | --- | --- | --- | --- | --- | --- |
| **Sample** | | **Gender** | | **Age** | **Weight** | | **Practice** | **Smoking time (years)** | **Last year averaged cig per day** | **Aternative practice (months)** | **Nicotine liquid refill concentration (mg/mL)** | **Consumed**  **volume (mL/day)** | **Vaping solution type**  **Free base/salts** | **Living with smokers** | | **Creatinine**  **(g L^-1^_urine_)** | |  |
| **Sample 01** | | Man | | 53 | 73 | | Vaper | 26 | 30 | 132 | 3 | 20 | Free base | No | | 2.48 | |  |
| **Sample 02** | | Woman | | 41 | 60 | | Vaper | 15 | 10 | 96 | 2 | 8 | Free base | Yes | | 1.06 | |  |
| **Sample 03** | | Woman | | 47 | 70 | | Vaper | 18 | 15 | 108 | 12 | 2 | Free base | No | | 1.98 | |  |
| **Sample 04** | | Woman | | 62 | 70 | | Smoker | 34 | 15 | - | - | - | - | Yes | | 1.44 | |  |
| **Sample 05** | | Woman | | 37 | 50 | | Vaper | 20 | 20 | 80 | 3 | 15 | Free base | No | | 1.85 | |  |
| **Sample 06** | | Man | | 50 | 130 | | Vaper | 30 | 50 | 120 | 3 | 7 | Free base | Yes | | 0.91 | |  |
| **Sample 07** | | Man | | 68 | 81 | | Non-smoker | - | - | - | - | - | - | Yes | | 0.87 | |  |
| **Sample 08** | | Man | | 44 | 63 | | Smoker | 25 | 15 | - | - | - | - | Yes | | 1.08 | |  |
| **Sample 09** | | Man | | 54 | 102 | | Non-smoker | - | - | - | - | - | - | - | | 0.90 | |  |
| **Sample 10** | | Man | | 50 | 88 | | Vaper | 25 | 20 | 132 | 3 | 20 | Free base | No | | 1.28 | |  |
| **Sample 11** | | Man | | 57 | 80 | | Vaper | 30 | 30 | 130 | 3 | 20 | Free base | Yes | | 0.73 | |  |
| **Sample 12** | | Woman | | 33 | 67 | | Non-smoker | - | - | - | - | - | - | Yes | | 0.59 | |  |
| **Sample 13** | | Woman | | 48 | 60 | | Vaper | 24 | 10 | 84 | 0 | 4 | - | No | | 0.64 | |  |
| **Sample 14** | | Woman | | 39 | 72 | | Vaper | 16 | 30 | 99 | 3/10 |  | Free base | No | | 0.96 | |  |
| **Sample 15** | | Woman | | 40 | 61 | | Vaper | 28 | 10 | 84 | 5 | 10 | Salts | Yes | | 1.23 | |  |
| **Sample 16** | | Woman | | 46 | 65 | | Vaper | 20 | 15 | 120 | 1 | 15 | Free base | No | | 2.56 | |  |
| **Sample 17** | | Man | | 50 | 72 | | Vaper | 26 | 20-30 | 91 | 3 | 10-15 | Free base | Yes | | 3.99*** | |  |
| **Sample 18** | | Man | | 54 | 107 | | Vaper | 30 | 40 | 108 | 6 | 10 | Free base | Yes | | 0.98 | |  |
| **Sample 19** | | Woman | | 74 | 70 | | Non-smoker | - | - | - | - | - | - | Yes | | 1.76 | |  |
| **Sample 20** | | Man | | 78 | 80 | | Smoker | 61 | 20 | - | - | - | - | No | | 0.68 | |  |
| **Sample 21** | | Woman | | 39 | 90 | | Vaper | 18 | 40 | 84 | 20 | 4 | Salts | No | | 1.59 | |  |
| **Sample 22** | | Woman | | 40 | 70 | | Vaper | 18 | 40 | 89 | 20 | 4 | Salts | No | | 0.56 | |  |
| **Sample 23** | | Man | | 51 | 88 | | Vaper | 25 | 20 | 144 | 3 | 20 | Free base | No | | 0.72 | |  |
| **Sample 24** | | Man | | 56 | 90 | | Vaper | 30 | 40 | 90 | 3 | 40 | Free base | Yes | | 0.40 | |  |
| **Sample 25** | | Man | | 45 | 150 | | Vaper | 25 | 50 | 108 | 3 | 10 | Free base | No | | 0.57 | |  |
| ***Table S1 (cont.).*** Sample population included in the study together with their gender, type of nicotine consumption system used, smoking time, use of an alternative practice to traditional tobacco and creatinine concentration in urine. | | | | | | | | | | | | | | | | | | |
| **Sample** | **Gender** | | **Age** | | | **Weight** | **Practice** | **Smoking time (years)** | **Last year averaged cig per day** | **Aternative practice (months)** | **Nicotine liquid refill concentration (mg/mL)** | **Consumed**  **volume (mL/day)** | **Vaping solution type**  **Free base/salts** | | **Living with smokers** | | **Creatinine**  **(g L^-1^_urine_)** | |
| **Sample 26** | Man | | 34 | | | 85 | Vaper | 11 | 24 | 96 | 3 | 10 | Free base | | Yes | | 0.77 | |
| **Sample 27** | Man | | 52 | | | 110 | Vaper | 23 | 6 | 86 | 0 | 2 | Free base | | Yes | | 0.77 | |
| **Sample 28** | Man | | 58 | | | 80 | Vaper | 30 | 30 | 142 | 3 | 20 | Free base | | Yes | | 0.78 | |
| **Sample 29** | Woman | | 28 | | | 52 | Non-smoker | - | - | - | - | - | - | | No | | 1.51 | |
| **Sample 30** | Woman | | 16 | | | 42 | Non-smoker | - | - | - | - | - | - | | No | | 0.67 | |
| **Sample 31** | Man | | 42 | | | 68 | Vaper | 8 | 8 | 96 | 18 | 7 | Free base | | No | | 0.45 | |
| **Sample 32** | Man | | 46 | | | 108 | Vaper | 20 | 15 | 72 | 3 | 18 | Free base | | No | | 0.75 | |
| **Sample 33** | Man | | 42 | | | 87 | Vaper | 15 | 30 | 72 | 3 | 15 | Free base | | Yes | | 1.41 | |
| **Sample 34** | Woman | | 46 | | | 74 | Vaper | 24 | 30 | 84 | 18 | 1 | Free base | | No | | 0.85 | |
| **Sample 35** | Woman | | 21 | | | 50 | Non-smoker | - | - | - | - | - | - | | Yes | | 0.35 | |
| **Sample 36** | Man | | 20 | | | 63 | Non-smoker | - | - | - | - | - | - | | No | | 0.87 | |
| **Sample 37** | Man | | 79 | | | 80 | Smoker | 62 | 20 | - | - | - | - | | No | | 1.04 | |
| **Sample 38** | Woman | | 75 | | | 70 | Non-smoker | - | - | - | - | - | - | | Yes | | 0.48 | |
| **Sample 39** | Woman | | 41 | | | 61 | Vaper | 28 | 10 | 84 | 5 | 10 | Salts | | Yes | | 0.59 | |
| **Sample 40** | Woman | | 26 | | | 56 | Vaper | 4 | 15 | 4 | 3 | 20 | Free base | | No | | 0.37 | |
| **Sample 41** | Man | | 58 | | | 80 | Vaper | 30 | 30 | 143 | 3 | 20 | Free base | | Yes | | 0.41 | |
| **Sample 42** | Man | | 51 | | | 88 | Vaper | 25 | 20 | 146 | 3 | 20 | Free base | | No | | 1.15 | |
| **Sample 43** | Woman | | 29 | | | 52 | Non-smoker | - | - | - | - | - | - | | - | | 1.25 | |
| **Sample 44** | Woman | | 64 | | | 59 | Smoker | 40 | 13 | - | - | - | - | | - | | 2.56 | |
| **Sample 45** | Woman | | 38 | | | 50 | Vaper | 20 | 20 | 92 | 3 | 15 | Free base | | No | | 0.37 | |
| **Sample 46** | Man | | 44 | | | 77 | Non-smoker | - | - | - | - | - | - | | - | | 2.05 | |
| **Sample 47** | Woman | | 34 | | | 67 | Non-smoker | - | - | - | - | - | - | | Yes | | 0.78 | |
| **Sample 48** | Woman | | 49 | | | 62 | Vaper | 15 | 40 | 96 | 1.5 | 15 | Free base | | No | | 1.99 | |

| ***Table S1 (cont.).*** Sample population included in the study together with their gender, type of nicotine consumption system used, smoking time, use of an alternative practice to traditional tobacco and creatinine concentration in urine. | | | | | | | | | | | | |
| --- | --- | --- | --- | --- | --- | --- | --- | --- | --- | --- | --- | --- |
| **Sample** | **Gender** | **Age** | **Weight** | **Practice** | **Smoking time (years)** | **Last year averaged cig per day** | **Aternative practice (months)** | **Nicotine liquid refill concentration (mg/mL)** | **Consumed**  **volume (mL/day)** | **Vaping solution type**  **Free base/salts** | **Living with smokers** | **Creatinine**  **(g L^-1^_urine_)** |
| **Sample 49** | Man | 60 | 78 | Smoker | 40 | 20 | - | - | - | - | Yes | 1.53 |
| **Sample 50** | Woman | 59 | 59 | Smoker | 40 | 10 | - | - | - | - | Yes | 1.92 |
| **Sample 51** | Man | 25 | 80 | Vaper | 8 | 20 | 36 | 2 | 20 | Free base | No | 3.38*** |
| **Sample 52** | Woman | 59 | 59 | Smoker | 40 | 10 | - | - | - | - | Yes | 0.76 |
| **Sample 53** | Man | 60 | 78 | Smoker | 40 | 20 | - | - | - | - | Yes | 1.38 |
| **Sample 54** | Man | 44 | 77 | Non-smoker | - | - | - | - | - | - | - | 1.55 |
| **Sample 55** | Woman | 46 | 74 | Vaper | 24 | 30 | 84 | 18 | 1 | Free base | No | 0.33 |
| **Sample 56** | Man | 42 | 87 | Vaper | 15 | 30 | 72 | 3 | 15 | Free base | Yes | 0.84 |
| **Sample 57** | Man | 46 | 108 | Vaper | 20 | 15 | 72 | 3 | 18 | Free base | No | 1.55 |
| **Sample 58** | Man | 42 | 68 | Vaper | 8 | 8 | 96 | 18 | 7 | Free base | No | 0.72 |
| **Sample 59** | Woman | 16 | 42 | Non-smoker | - | - | - | - | - | - | No | 0.39 |
| **Sample 60** | Woman | 28 | 52 | Non-smoker | - | - | - | - | - | - | No | 0.63 |
| **Sample 61** | Man | 58 | 80 | Vaper | 30 | 30 | 142 | 3 | 20 | Free base | Yes | 1.65 |
| **Sample 62** | Man | 52 | 110 | Vaper | 23 | 6 | 86 | 0 | 2 | Free base | Yes | 1.34 |
| **Sample 63** | Man | 34 | 85 | Vaper | 11 | 24 | 96 | 3 | 10 | Free base | Yes | 1.63 |
| **Sample 64** | Man | 45 | 150 | Vaper | 25 | 50 | 108 | 3 | 10 | Free base | No | 1.02 |
| **Sample 65** | Man | 56 | 90 | Vaper | 30 | 40 | 90 | 3 | 40 | Free base | Yes | 0.53 |
| **Sample 66** | Man | 51 | 88 | Vaper | 25 | 20 | 144 | 3 | 20 | Free base | No | 1.25 |
| **Sample 67** | Woman | 40 | 70 | Vaper | 18 | 40 | 89 | 20 | 4 | Salts | No | 0.86 |
| **Sample 68** | Woman | 39 | 90 | Vaper | 18 | 40 | 84 | 20 | 4 | Salts | No | 1.61 |
| **Sample 69** | Man | 78 | 80 | Smoker | 62 | 20 | - | - | - | - | No | 0.72 |
| **Sample 70** | Woman | 74 | 70 | Non-smoker | - | - | - | - | - | - | Yes | 2.09 |
| **Sample 71** | Man | 54 | 107 | Vaper | 30 | 40 | 108 | 6 | 10 | Free base | Yes | 0.92 |
| **Sample 72** | Man | 50 | 72 | Vaper | 26 | 20-30 | 91 | 3 | 10-15 | Free base | Yes | 3.76*** |
| **Sample 73** | Woman | 46 | 65 | Vaper | 20 | 15 | 120 | 1 | 15 | Free base | No | 2.38 |

| ***Table S1 (cont.).*** Sample population included in the study together with their gender, type of nicotine consumption system used, smoking time, use of an alternative practice to traditional tobacco and creatinine concentration in urine. | | | | | | | | | | | | |
| --- | --- | --- | --- | --- | --- | --- | --- | --- | --- | --- | --- | --- |
| **Sample** | **Gender** | **Age** | **Weight** | **Practice** | **Smoking time (years)** | **Last year averaged cig per day** | **Aternative practice (months)** | **Nicotine liquid refill concentration (mg/mL)** | **Consumed**  **volume (mL/day)** | **Vaping solution type**  **Free base/salts** | **Living with smokers** | **Creatinine**  **(g L^-1^_urine_)** |
| **Sample 74** | Woman | 40 | 61 | Vaper | 28 | 10 | 84 | 5 | 10 | Salts | Yes | 1.04 |
| **Sample 75** | Woman | 39 | 72 | Vaper | 16 | 30 | 99 | 3 | 10 | Free base | No | 0.91 |
| **Sample 76** | Woman | 48 | 60 | Vaper | 24 | 10 | 84 | 0 | 4 | - | No | 0.57 |
| **Sample 77** | Woman | 33 | 67 | Non-smoker | - | - | - | - | - | - | Yes | 0.68 |
| **Sample 78** | Man | 57 | 80 | Vaper | 30 | 30 | 130 | 3 | 20 | Free base | Yes | 0.62 |
| **Sample 79** | Man | 50 | 88 | Vaper | 25 | 20 | 132 | 3 | 20 | Free base | No | 1.26 |
| **Sample 80** | Man | 54 | 102 | Non-smoker | - | - | - | - | - | - | - | 0.88 |
| **Sample 81** | Man | 44 | 63 | Smoker | 25 | 15 | - | - | - | - | Yes | 1.35 |
| **Sample 82** | Man | 68 | 81 | Non-smoker | - | - | - | - | - | - | Yes | 1.88 |
| **Sample 83** | Man | 50 | 130 | Vaper | 30 | 50 | 120 | 3 | 7 | Free base | Yes | 0.92 |
| **Sample 84** | Woman | 37 | 50 | Vaper | 20 | 20 | 80 | 3 | 15 | Free base | No | 1.90 |
| **Sample 85** | Woman | 62 | 70 | Smoker | 34 | 15 | - | - | - | - | Yes | 1.59 |
| **Sample 86** | Woman | 47 | 70 | Vaper | 18 | 15 | 108 | 12 | 2 | Free base | No | 2.01 |
| **Sample 87** | Woman | 41 | 60 | Vaper | 15 | 10 | 96 | 2 | 8 | Free base | Yes | 1.03 |
| **Sample 88** | Man | 53 | 73 | Vaper | 26 | 30 | 132 | 3 | 20 | Free base | No | 2.46 |
| **Sample 89** | Woman | 64 | 59 | Smoker | 40 | 13 | - | - | - | - | - | 0.32 |
| **Sample 90** | Woman | 29 | 52 | Non-smoker | - | - | - | - | - | - | - | 2.60 |
| **Sample 91** | Man | 51 | 88 | Vaper | 25 | 20 | 146 | 3 | 20 | Free base | No | 1.84 |
| **Sample 92** | Man | 58 | 80 | Vaper | 30 | 30 | 143 | 3 | 20 | Free base | Yes | 1.18 |
| **Sample 93** | Woman | 26 | 56 | Vaper | 4 | 15 | 4 | 3 | 20 | Free base | No | 0.50 |
| **Sample 94** | Woman | 41 | 61 | Vaper | 28 | 10 | 84 | 5 | 10 | Salts | Yes | 0.43 |
| **Sample 95** | Woman | 75 | 70 | Non-smoker | - | - | - | - | - | - | Yes | 0.66 |

| ***Table S1 (cont.).*** Sample population included in the study together with their gender, type of nicotine consumption system used, smoking time, use of an alternative practice to traditional tobacco and creatinine concentration in urine. | | | | | | | | | | | | |
| --- | --- | --- | --- | --- | --- | --- | --- | --- | --- | --- | --- | --- |
| **Sample** | **Gender** | **Age** | **Weight** | **Practice** | **Smoking time (years)** | **Last year averaged cig per day** | **Aternative practice (months)** | **Nicotine liquid refill concentration (mg/mL)** | **Consumed**  **volume (mL/day)** | **Vaping solution type**  **Free base/salts** | **Living with smokers** | **Creatinine**  **(g L^-1^_urine_)** |
| **Sample 96** | Man | 79 | 80 | Smoker | 62 | 20 | - | - | - | - | No | 0.61 |
| **Sample 97** | Woman | 21 | 50 | Non-smoker | - | - | - | - | - | - | Yes | 0.85 |
| **Sample 98** | Man | 20 | 63 | Non-smoker | - | - | - | - | - | - | No | 1.18 |
| **Sample 99** | Woman | 49 | 62 | Vaper | 15 | 40 | 96 | 1.5 | 15 | Free base | No | 0.36 |
| **Sample 100** | Woman | 34 | 67 | Non-smoker | - | - | - | - | - | - | Yes | 1.63 |
| **Sample 101** | Man | 44 | 77 | Non-smoker | - | - | - | - | - | - | - | 0.58 |
| **Sample 102** | Woman | 38 | 50 | Vaper | 20 | 20 | 92 | 3 | 15 | Free base | No | 2.83 |
| ***: value higher than the 3 g L^-1^ recommended maximum | | | | | | | | | | | | |

| ***Table S2.***  Metabolites, ionization mode and transitions m/z employed for the metabolites quantification by LC-MS/MS. | | | | |
| --- | --- | --- | --- | --- |
| **Analyte** | **Ionization** | **Precursor ion** | **1^st^ fragment**  **(quantification ion)** | **2^nd^ fragment**  **(qualifier ion)** |
| Nicotine | ESI^+^ | 163.212 | 117.042 (CE 25.89)*^a^* | 98.958 (CE 11.66)*^a^* |
| Nicotine-d_4_ | ESI^+^ | 167.212 | 134.125 (CE 20.54)*^a^* | 110.113 (CE 15.99)*^a^* |
| Cotinine | ESI^+^ | 177.162 | 145.042 (CE 7.99)*^a^* | 80.042 (CE 23.87)*^a^* |
|  | | | | |
| **Analyte** | **Ionization** | **Precursor Ion** | **Quantification ion** | **Confirmation Ion** |
| 3HPMA | ESI^-^ | 220.04 | 91.04 (CE 13.72)*^a^* | 89 (CE 21.51) *^a^* |
| CEMA | ESI^-^ | 214.95 | 162.04 (CE 7.78) *^a^* | 86.04 (CE 12.96) *^a^* |
| CMEMA | ESI^+^ | 250.09 | 204.07 (CE 9.59) *^a^* | 232.13 (CE 7.15) *^a^* |
| DHBMA | ESI^-^ | 250.04 | 120.97 (CE 14.31) *^a^* | 75.04 (CE 22.99) *^a^* |
| HMPMA | ESI^-^ | 234.00 | 105 (CE 13.4) *^a^* | 103.11 (CE 21.6) *^a^* |
| MHBMA | ESI^-^ | 231.95 | 103.04 (CE 12.16) *^a^* | 128.04 (CE 8.66) *^a^* |
| *^a^*: CE (collision energy (V)) | | | | |

| ***Table S3.*** Analytical features of nicotine, cotinine, acrylonitrile and crotonaldehyde determination procedure (a) and recovery and RSD (%) values obtained for urine spiked samples (b). | | | | | |
| --- | --- | --- | --- | --- | --- |
| (a) | | | | | |
|  | **Slope x 10^3^** | **Intercept** | **R^2^** | **LOD*^a^* (ng mL^1^)** | **LOQ*^a^* (ng mL^-1^)** |
| **Nicotine** | 7.741 | 0.5566 | 0.991 | 47 | 144 |
| **Cotinine** | 14.73 | 0.3951 | 0.992 | 45 | 134 |
|  |  |  |  |  |  |
|  | **Slope (ng^-1^)** | **Intercept** | **R^2^** | **LOD*^a^* (ng mL^-1^)** | **LOQ*^a^* (ng mL^-1^)** |
| **Acrylonitrile** | 0.0391 | -0.0183 | 0.9951 | 0.33 | 1.01 |
| **Crotonaldehyde** | 0.2202 | -0.1910 | 0.993 | 1.4 | 4.6 |
| *^a^*: the limits of detection (LOD) and of quantification (LOQ) were determined from calibration expressions as 3 and 10 times the intercept divided by the slope, respectively | | | | | |
| (b) |  |  |  |  |  |
|  | **C _added_ (ng mL^-1^)** | | **Recovery (% ± s, n=3)** | | **RSD (%, n=3)** |
| **Nicotine** | 190 | | 104±3 | | 3 |
|  | 1400 | | 93±4 | | 4 |
|  | 3000 | | 101±5 | | 5 |
|  | 3800 | | 104±3 | | 3 |
| **Cotinine** | 150 | | 93±5 | | 6 |
|  | 750 | | 101±4 | | 4 |
|  | 1100 | | 95±4 | | 4 |
|  | 2250 | | 96±7 | | 7 |
|  | 3000 | | 97±9 | | 9 |
| **Acrylonitrile** | 25 | | 84±9 | | 11 |
|  | 100 | | 96±5 | | 5 |
|  | 200 | | 99±4 | | 4 |
| **Crotonaldehyde** | 6 | | 97±2 | | 2 |
|  | 25 | | 101±5 | | 4 |
|  | 50 | | 108±2 | | 2 |

| ***Table S4*.** Analytical features of DHBMA, MHBMA, CEMA, 3-HPMA, CMEMA and HMPMA determination procedure (a), and recovery and RSD (%) values obtained for urine spiked samples (b). | | | | | | | | | | | |
| --- | --- | --- | --- | --- | --- | --- | --- | --- | --- | --- | --- |
| (a) | | | | | | | | | | | |
|  | **Intercept** | | | **slope** | | **R^2^** | | **LOD*^a^* (ng mL^-1^)** | | **LOQ*^a^* (ng mL^-1^)** | |
| **DHBMA** | 0.92 | | | 1.82 | | 0.9901 | | 3.0 | | 9.0 | |
| **MHBMA** | 0.078 | | | 1.70 | | 0.9984 | | 3.3 | | 11.0 | |
| **CEMA** | 1.26 | | | 11.02 | | 0.9927 | | 3.3 | | 11.0 | |
| **3-HPMA** | 0.029 | | | 0.54 | | 0.9974 | | 2.9 | | 9.0 | |
| **CMEMA** | 0.062 | | | 0.57 | | 0.9965 | | 2.8 | | 9.5 | |
| **HMPMA** | 0.038 | | | 1.3 | | 0.9948 | | 3.1 | | 10.3 | |
| *^a^*: the limits of detection (LOD) and of quantification (LOQ) were determined from calibration expressions as 3 and 10 times the intercept divided by the slope, respectively | | | | | | | | | | | |
| (b) | | | | | | | | | | | |
|  | | **Recovery (% ± s, n=3)** | | | | | | | | | |
| **C _added_ (ng mL^-1^)** | | **DHBMA** | **MHBMA** | | **CEMA** | | **3-HPMA** | | **CMEMA** | | **HMPMA** |
| 10 | | 89±2 (2) | 90±5 (5) | | 87±5 (6) | | 91±3 (3) | | 88±4 (4) | | 93±2 (2) |
| 50 | | 91±4 (4) | 94±2 (2) | | 94±1 (1) | | 97±4 (4) | | 99±2 (2) | | 91±4 (4) |
| 100 | | 95±1 (1) | 103±6 (6) | | 98±3 (3) | | 94±1 (1) | | 97±3 (3) | | 98±5 (5) |
| 250 | | 101±3 (3) | 96±3 (3) | | 97±5 (5) | | 101±6 (6) | | 104±3 (3) | | 97±1 (1) |
| 500 | | 98±3 (3) | 95±1 (1) | | 100±2 (2) | | 100±2 (2) | | 97±3 (3) | | 96±2 (2) |
| 750 | | 99±1 (1) | 104±7 (7) | | 95±4 (4) | | 97±7 (7) | | 100±1 (1) | | 103±4 (4) |
| Note: RSD (%) values are indicated in brackets. | | | | | | | | | | | |

| ***Table S5*.** Nicotine and cotinine concentration, expressed as µg g^-1^_creatinine_, in analyzed urine samples. | | | | | |
| --- | --- | --- | --- | --- | --- |
|  | | | **[concentration in urine (µg g^-1^_creatinine_)]** | |  |
|  |  | **Living with smokers** | **Nicotine** | **Cotinine** | **Nicotine/cotinine ratio** |
| **Sample 01** | Vaper | No | 3565 | 3990 | 0.89 |
| **Sample 02** | Vaper | Yes | 1263 | 1770 | 0.71 |
| **Sample 03** | Vaper | No | 2160 | 3530 | 0.61 |
| **Sample 04** | Smoker | Yes | 880 | 1120 | 0.79 |
| **Sample 05** | Vaper | No | 2630 | 1290 | 2.04 |
| **Sample 06** | Vaper | Yes | 1364 | 910 | 1.50 |
| **Sample 07** | Non-smoker | No | *n.d* | *n.d* | 0.00 |
| **Sample 08** | Smoker | Yes | 3565 | 2750 | 1.30 |
| **Sample 09** | Non-smoker | No | *n.d* | *n.d* | 0.00 |
| **Sample 10** | Vaper | No | 1430 | 1825 | 0.78 |
| **Sample 11** | Vaper | Yes | 910 | 2580 | 0.35 |
| **Sample 12** | Non-smoker | Yes | *n.d* | *n.d* | 0.00 |
| **Sample 13** | Vaper | No | 80 | 36 | 2.22 |
| **Sample 14** | Vaper | No | 1235 | 4430 | 0.28 |
| **Sample 15** | Vaper | Yes | 630 | 5320 | 0.12 |
| **Sample 16** | Vaper | No | 5795 | 6110 | 0.95 |
| **Sample 17** | Vaper | Yes | 9400 | 3430 | 2.74 |
| **Sample 18** | Vaper | Yes | 160 | 340 | 0.47 |
| **Sample 19** | Non-smoker | Yes | 84 | 74 | 1.14 |
| **Sample 20** | Smoker | No | 380 | 560 | 0.68 |
| **Sample 21** | Vaper | No | 1840 | 4745 | 0.39 |
| **Sample 22** | Vaper | No | 483 | 6935 | 0.07 |
| **Sample 23** | Vaper | No | 1158 | 3230 | 0.36 |
| **Sample 24** | Vaper | Yes | 1835 | 634 | 2.89 |
| **Sample 25** | Vaper | No | 3045 | 6118 | 0.50 |
| **Sample 26** | Vaper | Yes | 3974 | 4316 | 0.92 |
| **Sample 27** | Vaper | Yes | *n.d* | *n.d* | 0.00 |
| **Sample 28** | Vaper | Yes | 2496 | 6746 | 0.37 |
| **Sample 29** | Non-smoker | No | *n.d* | *n.d* | 0.00 |
| **Sample 30** | Non-smoker | No | *n.d* | *n.d* | 0.00 |
| **Sample 31** | Vaper | No | 7587 | 17820 | 0.43 |
| **Sample 32** | Vaper | No | 1052 | 2479 | 0.42 |
| **Sample 33** | Vaper | Yes | 865 | 2981 | 0.29 |
| **Sample 34** | Vaper | No | 346 | 1271 | 0.27 |
| **Sample 35** | Non-smoker | Si | *n.d* | *n.d* | 0.00 |
| **Sample 36** | Non-smoker | No | *n.d* | *n.d* | 0.00 |
| **Sample 37** | Smoker | No | 237 | 2832 | 0.08 |
| **Sample 38** | Non-smoker | Si | *n.d* | *n.d* | 0.00 |
| **Sample 39** | Vaper | Si | 109 | 1172 | 0.09 |
| *n.d.:* non-detected | | | | |  |
| ***Table S5 (Cont.)*.** Nicotine and cotinine concentration, expressed as µg g^-1^_creatinine_, in analyzed urine samples. | | | | |  |
|  | | | **[concentration in urine (µg g^-1^_creatinine_)]** | |  |
|  |  | **Living with smokers** | **Nicotine** | **Cotinine** | **Nicotine/cotinine ratio** |
| **Sample 40** | Vaper | No | 3487 | 5736 | 0.61 |
| **Sample 41** | Vaper | Yes | 1847 | 6971 | 0.26 |
| **Sample 42** | Vaper | No | 1529 | 7769 | 0.20 |
| **Sample 43** | Non-smoker | No | *n.d* | *n.d* | 0.00 |
| **Sample 44** | Smoker | No | 1643 | 2470 | 0.67 |
| **Sample 45** | Vaper | No | 1139 | 5887 | 0.19 |
| **Sample 46** | Non-smoker | No | *n.d* | *n.d* | 0.00 |
| **Sample 47** | Non-smoker | Yes | *n.d* | *n.d* | 0.00 |
| **Sample 48** | Vaper | No | 492 | 2128 | 0.23 |
| **Sample 49** | Smoker | Yes | 501 | 2495 | 0.20 |
| **Sample 50** | Smoker | Yes | 60 | 276 | 0.22 |
| **Sample 51** | Vaper | No | 364 | 302 | 1.21 |
| **Sample 52** | Smoker | Yes | 2279 | 5463 | 0.42 |
| **Sample 53** | Smoker | Yes | 560 | 350 | 1.60 |
| **Sample 54** | Non-smoker | No | *n.d* | *n.d* | 0.00 |
| **Sample 55** | Vaper | No | 327 | 1274 | 0.26 |
| **Sample 56** | Vaper | Yes | 823 | 2842 | 0.29 |
| **Sample 57** | Vaper | No | 1079 | 2030 | 0.53 |
| **Sample 58** | Vaper | No | 7364 | 15942 | 0.46 |
| **Sample 59** | Non-smoker | No | *n.d* | *n.d* | 0.00 |
| **Sample 60** | Non-smoker | No | *n.d* | *n.d* | 0.00 |
| **Sample 61** | Vaper | Yes | 2446 | 6656 | 0.37 |
| **Sample 62** | Vaper | Yes | *n.d* | *n.d* | 0.00 |
| **Sample 63** | Vaper | Yes | 3272 | 4134 | 0.79 |
| **Sample 64** | Vaper | No | 2918 | 5780 | 0.50 |
| **Sample 65** | Vaper | Yes | 1861 | 682 | 2.73 |
| **Sample 66** | Vaper | No | 1136 | 3298 | 0.34 |
| **Sample 67** | Vaper | No | 499 | 6883 | 0.07 |
| **Sample 68** | Vaper | No | 1865 | 4821 | 0.39 |
| **Sample 69** | Smoker | No | 367 | 686 | 0.53 |
| **Sample 70** | Non-smoker | Yes | 90 | 64 | 1.41 |
| **Sample 71** | Vaper | Yes | 200 | 431 | 0.46 |
| **Sample 72** | Vaper | Yes | 9543 | 3547 | 2.69 |
| **Sample 73** | Vaper | No | 5406 | 7814 | 0.69 |
| **Sample 74** | Vaper | Yes | 664 | 5332 | 0.12 |
| **Sample 75** | Vaper | No | 1227 | 4444 | 0.28 |
| **Sample 76** | Vaper | No | *n.d* | 36 | 0.00 |
| **Sample 77** | Non-smoker | Yes | *n.d* | *n.d* | 0.00 |
| **Sample 78** | Vaper | Yes | 974 | 2288 | 0.43 |
| *n.d.: non-detected* | | | | |  |
| ***Table S5 (Cont.)*.** Nicotine and cotinine concentration, expressed as µg g^-1^_creatinine_, in analyzed urine samples. | | | | | |
|  | | | **[concentration in urine (µg g^-1^_creatinine_)]** | |  |
|  |  | **Living with smokers** | **Nicotine** | **Cotinine** | **Nicotine/cotinine ratio** |
| **Sample 79** | Vaper | No | 1702 | 1890 | 0.90 |
| **Sample 80** | Non-smoker | No | *n.d* | *n.d* | 0.00 |
| **Sample 81** | Smoker | Yes | 3389 | 2879 | 1.18 |
| **Sample 82** | Non-smoker | No | *n.d* | *n.d* | 0.00 |
| **Sample 83** | Vaper | Yes | 1571 | 917 | 1.71 |
| **Sample 84** | Vaper | No | 2332 | 1237 | 1.89 |
| **Sample 85** | Smoker | Yes | 648 | 1148 | 0.56 |
| **Sample 86** | Vaper | No | 1866 | 3598 | 0.52 |
| **Sample 87** | Vaper | Yes | 1159 | 1826 | 0.63 |
| **Sample 88** | Vaper | No | 3049 | 2966 | 1.03 |
| **Sample 89** | Smoker | No | 1650 | 2879 | 0.57 |
| **Sample 90** | Non-smoker | No | *n.d* | *n.d* | 0.00 |
| **Sample 91** | Vaper | No | 1867 | 7791 | 0.24 |
| **Sample 92** | Vaper | Yes | 1651 | 6716 | 0.25 |
| **Sample 93** | Vaper | No | 3584 | 5401 | 0.66 |
| **Sample 94** | Vaper | Yes | 150 | 1163 | 0.13 |
| **Sample 95** | Non-smoker | Yes | *n.d* | *n.d* | 0.00 |
| **Sample 96** | Smoker | No | 286 | 2564 | 0.11 |
| **Sample 97** | Non-smoker | Yes | *n.d* | *n.d* | 0.00 |
| **Sample 98** | Non-smoker | No | *n.d* | *n.d* | 0.00 |
| **Sample 99** | Vaper | No | 478 | 2180 | 0.22 |
| **Sample 100** | Non-smoker | Yes | *n.d* | *n.d* | 0.00 |
| **Sample 101** | Non-smoker | No | *n.d* | *n.d* | 0.00 |
| **Sample 102** | Vaper | No | 1284 | 5892 | 0.22 |
| *n.d: non-detected* | | | | | |

| \| ***Table S6*.** Metabolite concentrations, expressed as µg g^-1^_creatinine_, in analyzed urine samples. \| \| --- \| | | | | | | |
| --- | --- | --- | --- | --- | --- | --- | --- |
|  | **[concentration in urine (µg g^-1^_creatinine_)]** | | | | | |
|  | **3HPMA** | **CEMA** | **HMPMA** | **MHBMA** | **CMEMA** | **DHBMA** |
| **Sample 01** | 172 | n.d. | 165 | 41 | 333 | 276 |
| **Sample 02** | 1289 | n.d. | 391 | 129 | 1618 | 480 |
| **Sample 03** | 236 | n.d. | 321 | 58 | 213 | 554 |
| **Sample 04** | 940 | 126 | 1355 | 46 | 817 | 481 |
| **Sample 05** | 310 | n.d. | 147 | 44 | 1015 | 274 |
| **Sample 06** | 6156 | 193 | 3527 | 298 | 5493 | 959 |
| **Sample 07** | 374 | n.d. | 542 | 87 | 696 | 945 |
| **Sample 08** | 851 | 95 | 1332 | 66 | 343 | 241 |
| **Sample 09** | 1113 | n.d. | 258 | 119 | 748 | 332 |
| **Sample 10** | 2066 | n.d. | 418 | 119 | 4027 | 472 |
| **Sample 11** | 878 | n.d. | 280 | 97 | 4006 | 299 |
| **Sample 12** | 336 | n.d. | 136 | 91 | 114 | 144 |
| **Sample 13** | 980 | n.d. | 311 | 102 | 1057 | 152 |
| **Sample 14** | 284 | n.d. | 241 | 65 | 375 | 351 |
| **Sample 15** | 819 | n.d. | 242 | 70 | 3845 | 346 |
| **Sample 16** | 1566 | n.d. | 379 | 103 | 5663 | 562 |
| **Sample 17** | 235 | n.d. | 474 | 52 | 893 | 451 |
| **Sample 18** | 476 | n.d. | 1032 | 123 | 1234 | 193 |
| **Sample 19** | 235 | n.d. | 314 | 49 | 409 | 394 |
| **Sample 20** | 1924 | 184 | 2625 | 166 | 2190 | 545 |
| **Sample 21** | 727 | n.d. | 906 | 130 | 10989 | 513 |
| **Sample 22** | 1048 | n.d. | 1482 | 317 | 3705 | 537 |
| **Sample 23** | 2868 | n.d. | 536 | 144 | 2117 | 766 |
| **Sample 24** | 737 | n.d. | 1261 | 270 | 924 | 545 |
| **Sample 25** | 1288 | n.d. | 1930 | 185 | 3868 | 950 |
| **Sample 26** | 1060 | n.d. | 481 | 151 | 5596 | 368 |
| **Sample 27** | 530 | n.d. | 349 | 157 | 1253 | 432 |
| **Sample 28** | 494 | n.d. | 399 | 55 | 968 | 299 |
| **Sample 29** | 534 | n.d. | 319 | 101 | 3020 | 294 |
| **Sample 30** | 927 | n.d. | 223 | 161 | 1361 | 177 |
| **Sample 31** | 418 | n.d. | 239 | 84 | 630 | 331 |
| **Sample 32** | 16947 | n.d. | 397 | 95 | 1449 | 386 |
| **Sample 33** | 645 | n.d. | 480 | 95 | 1368 | 404 |
| **Sample 34** | 1064 | n.d. | n.d. | 232 | 475 | 71 |
| **Sample 35** | 483 | n.d. | 435 | 91 | 443 | 376 |
| **Sample 36** | 252 | n.d. | 228 | 47 | 251 | 313 |
| **Sample 37** | 4203 | 265 | 5330 | 110 | 6102 | 855 |
| **Sample 38** | 643 | n.d. | 1825 | 90 | 912 | 501 |
| **Sample 39** | 757 | n.d. | 662 | 152 | 1776 | 256 |
| n.d.: non-detected | | | | | | |

| \| ***Table S6 (cont.)*.** Metabolite concentrations, expressed as µg g^-1^_creatinine_, in analyzed urine samples. \| \| --- \| | | | | | | |
| --- | --- | --- | --- | --- | --- | --- | --- |
|  | **[concentration in urine (µg g^-1^_creatinine_)]** | | | | | |
|  | **3HPMA** | **CEMA** | **HMPMA** | **MHBMA** | **CMEMA** | **DHBMA** |
| **Sample 40** | 766 | n.d. | 369 | 144 | 4717 | 469 |
| **Sample 41** | 722 | n.d. | 368 | 65 | 4064 | 336 |
| **Sample 42** | 714 | n.d. | 491 | 111 | 1630 | 296 |
| **Sample 43** | 207 | n.d. | 201 | 97 | 725 | 219 |
| **Sample 44** | 1766 | 234 | 3123 | 123 | 1490 | 811 |
| **Sample 45** | 203 | n.d. | 225 | 31 | 515 | 288 |
| **Sample 46** | 286 | n.d. | 159 | 70 | 398 | 183 |
| **Sample 47** | 167 | n.d. | 220 | 35 | 300 | 490 |
| **Sample 48** | 1865 | n.d. | 397 | 200 | 1890 | 566 |
| **Sample 49** | 1294 | 255 | 2798 | 66 | 462 | 161 |
| **Sample 50** | 2860 | 41 | 881 | 52 | 116 | 53 |
| **Sample 51** | 868 | n.d. | 314 | 88 | 707 | 93 |
| **Sample 52** | 2675 | 60 | 4315 | 127 | 188 | 351 |
| **Sample 53** | 3106 | 370 | 4527 | 104 | 838 | 328 |
| **Sample 54** | 2729 | n.d. | 748 | 194 | 1452 | 185 |
| **Sample 55** | 1322 | n.d. | n.d. | 304 | 363 | 58 |
| **Sample 56** | 799 | n.d. | 502 | 75 | 1593 | 469 |
| **Sample 57** | 20980 | n.d. | 314 | 81 | 1517 | 325 |
| **Sample 58** | 425 | n.d. | 106 | 83 | 622 | 310 |
| **Sample 59** | 1160 | n.d. | 578 | 180 | 1367 | 168 |
| **Sample 60** | 769 | n.d. | 200 | 184 | 2534 | 257 |
| **Sample 61** | 629 | n.d. | 611 | 94 | 625 | 238 |
| **Sample 62** | 325 | n.d. | 32 | 159 | 1193 | 471 |
| **Sample 63** | 633 | n.d. | 249 | 157 | 4215 | 352 |
| **Sample 64** | 783 | n.d. | 1343 | 172 | 2223 | 961 |
| **Sample 65** | 575 | n.d. | 890 | 396 | 886 | 419 |
| **Sample 66** | 1938 | n.d. | 223 | 139 | 2603 | 627 |
| **Sample 67** | 884 | n.d. | 1136 | 337 | 2627 | 580 |
| **Sample 68** | 872 | n.d. | 350 | 185 | 9142 | 517 |
| **Sample 69** | 1780 | 154 | 3185 | 141 | 1916 | 588 |
| **Sample 70** | 255 | n.d. | 307 | 55 | 487 | 396 |
| **Sample 71** | 579 | n.d. | 1001 | 125 | 1470 | 124 |
| **Sample 72** | 365 | n.d. | 620 | 48 | 858 | 419 |
| **Sample 73** | 2583 | n.d. | 455 | 105 | 6379 | 534 |
| **Sample 74** | 1549 | n.d. | 182 | 72 | 3720 | 273 |
| **Sample 75** | 365 | n.d. | 235 | 64 | 366 | 370 |
| **Sample 76** | 1557 | n.d. | 68 | 109 | 880 | 188 |
| **Sample 77** | 326 | n.d. | 253 | 98 | 126 | 152 |
| **Sample 78** | 1232 | n.d. | 217 | 81 | 2147 | 300 |
| n.d.: non-detected | | | | | | |

| \| ***Table S6 (cont.)*.** Metabolite concentrations, expressed as µg g^-1^_creatinine_, in analyzed urine samples. \| \| --- \| | | | | | | |
| --- | --- | --- | --- | --- | --- | --- | --- |
|  | **[concentration in urine (µg g^-1^_creatinine_)]** | | | | | |
|  | **3HPMA** | **CEMA** | **HMPMA** | **MHBMA** | **CMEMA** | **DHBMA** |
| **Sample 79** | 3037 | n.d. | 270 | 117 | 3246 | 443 |
| **Sample 80** | 1414 | n.d. | 125 | 142 | 713 | 348 |
| **Sample 81** | 735 | 67 | 980 | 63 | 329 | 251 |
| **Sample 82** | 401 | n.d. | 446 | 75 | 642 | 977 |
| **Sample 83** | 9554 | 190 | 5388 | 202 | 4551 | 924 |
| **Sample 84** | 401 | n.d. | 11 | 88 | 614 | 280 |
| **Sample 85** | 1283 | 125 | 1728 | 37 | 888 | 407 |
| **Sample 86** | 316 | n.d. | 292 | 85 | 216 | 460 |
| **Sample 87** | 1936 | n.d. | 246 | 137 | 1789 | 485 |
| **Sample 88** | 225 | n.d. | 112 | 49 | 310 | 110 |
| **Sample 89** | 2544 | 266 | 3690 | 147 | 1070 | 791 |
| **Sample 90** | 271 | n.d. | 181 | 86 | 503 | 151 |
| **Sample 91** | 888 | n.d. | 461 | 125 | 974 | 300 |
| **Sample 92** | 1185 | n.d. | 385 | 69 | 3913 | 312 |
| **Sample 93** | 696 | n.d. | 226 | 155 | 3373 | 414 |
| **Sample 94** | 848 | n.d. | 212 | 185 | 1153 | 208 |
| **Sample 95** | 709 | n.d. | 1829 | 92 | 918 | 600 |
| **Sample 96** | 4995 | 266 | 5650 | 141 | 6047 | 769 |
| **Sample 97** | 621 | n.d. | 392 | 86 | 450 | 299 |
| **Sample 98** | 302 | n.d. | 76 | 49 | 352 | 374 |
| **Sample 99** | 2669 | n.d. | 453 | 166 | 1341 | 570 |
| **Sample 100** | 228 | n.d. | 168 | 35 | 342 | 350 |
| **Sample 101** | 357 | n.d. | 251 | 71 | 380 | 177 |
| **Sample 102** | 214 | n.d. | 180 | 35 | 550 | 220 |
| n.d.: non-detected | | | | | | |

***Figure S1*.** Universitat de Valencia ethics committee approval for the human specimens collected.


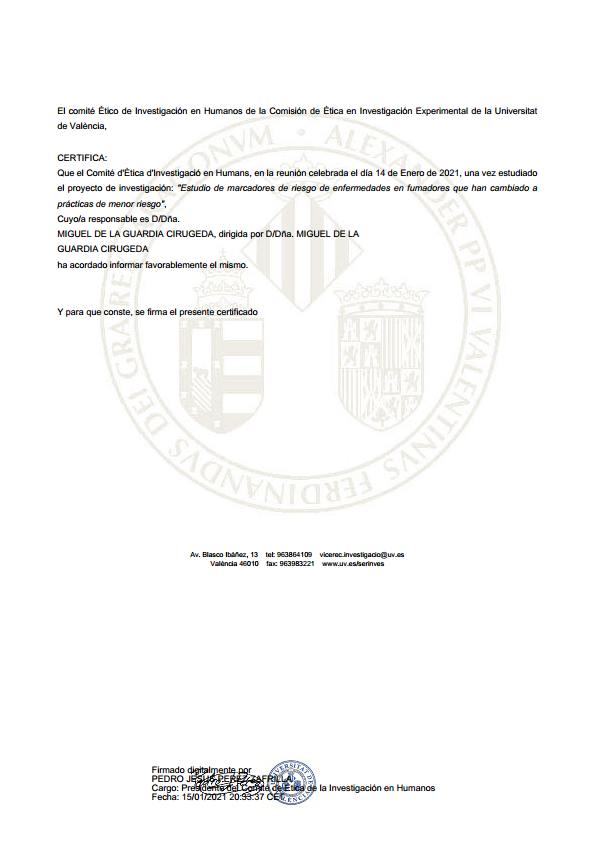


***Figure S1 (Cont.)*.** Universitat de Valencia ethics committee approval for the human specimens collected.


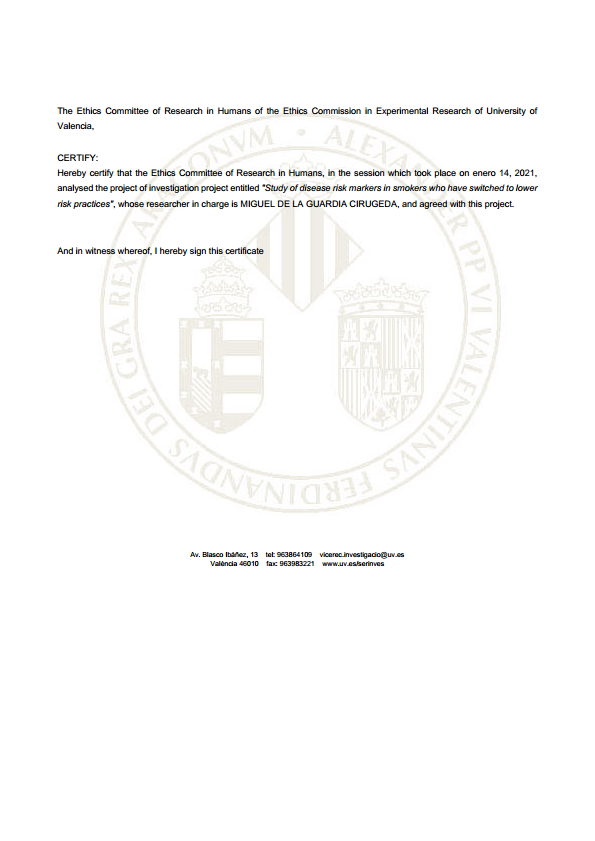

Supplement: Supplementary file 1 — Supplementary file1 (DOCX 540 KB) [file 216_2023_4943_MOESM1_ESM.docx]
